# Supplementary material for: Three Perspectives on the Experience of Support for Family Caregivers in First Nations Communities
Source: Diseases. 2023 Mar 8;11(1):47. doi: 10.3390/diseases11010047 (PMC10047389; doi:10.3390/diseases11010047)
Supplement: Supplementary file 1 [file diseases-11-00047-s001.zip › diseases-2234820-supplementary.pdf]

What are the educational needs of First and Metis Nations Healthcare Providers to prepare them to support First Nations and Metis Family Caregivers?

Focus Groups Semi-Structured Interview Guide

Methodology: We are using Participatory Action Research to guide interviews and interview analysis. We will review the interview transcript and adjust the interview guide as we learn how research participants make sense of their experiences and we make analytic sense of their meanings.

Introduction:

Thank you for taking part in this interview.

My name is Amber Ward. The study team is led by Dr. Jasneet Parmar from the University of Alberta. Dr. Parmar is a family and care of the elderly physician, so she understands healthcare and works with family caregivers. However, there is little research on how to support First Nations family caregivers.

Overview:

Alberta's family caregivers provide about 90% of the care to children, adults, and older adults with illness, disabilities, and frailty from aging. Health providers are well-positioned to support family caregivers, yet we know almost nothing about what the healthcare providers need to support First Nations family caregivers or what First Nations family caregivers expect from health providers.

We realize you are busy, and we appreciate your time. We expect the interview will take about 45 to 60 minutes.

We are recording the interview. The recordings will be kept in an encrypted folder on a password-protected University of Alberta computer safely in a locked facility until they are transcribed word for word. Any information that would allow you or your work setting to be identified will be removed from the transcriptions.

Despite being recorded, I would like to assure you in any reports or academic articles, you will not be identified. May I tape the discussion to facilitate its recollection? (If yes, switch on the recorder).

Have you read the information and consent form? Do we have your verbal consent to participate in this interview?

Guiding questions for participants:

Icebreaker questions

1. First, tell me about First Nations caregivers in this community.

   Probes:
   - Who needs care? children, adults, elders
   - Is there a traditional Cree view of caregiving?

What are the educational needs of First and Metis Nations Healthcare Providers to prepare them to support First Nations and Metis Family Caregivers?

2. Are you a caregiver? If caregiver: Can you tell me as little or as much as you want to tell me about your caregiving.

If not a caregiver: What is your role with the First Nations caregivers in this community? Can you tell me how you work with family caregivers here on the First Nation?

3. **Family Caregivers:** What makes it easy or hard for you to care? **Health Providers or Leaders:** What makes it easy or hard to care to family caregivers?

   Probes:
   - What works well?
   - What are the barriers? What makes it hard? Hours of respite, closures on weekend continuity of care. Scope of practice, Life skills versus what management

Continuing questions

4. If there was something that should be done to help support Aboriginal family caregivers?

   Probe:

   - What would that look like?

5. **Family Caregivers:** What knowledge and skills do health and community providers need to support family caregivers? **Health Providers:** In your experience, does your education and training equip you with the knowledge and skills to support family caregivers? When you are thinking about continuing education, what might be helpful to learn about supporting family caregivers?"

Final question:

6. We have asked you lots of questions, what advice would you give us about the kind of support health care providers in this community need to support family caregivers?

End of interview:

Thank you for participating. You have provided us with very thoughtful information. If there is anything else, you would like to tell us please email us.
